# Intraocular Pharmacokinetics and Safety of Subretinal Injection Compared with Intravitreal Application of Conbercept in Vitrectomized Rabbit Eyes

**DOI:** 10.1155/2020/2674780

**Published:** 2020-03-23

**Authors:** Teng-teng Yao, Xiao-liang Jin, Yuan Yang, Yi-xiao Wang, Ya-li Zhou, Fang-lin He, Zhao-yang Wang

**Affiliations:** ^1^Department of Ophthalmology, Shanghai Ninth People's Hospital, Shanghai Jiaotong University School of Medicine, Shanghai, China; ^2^Shanghai Key Laboratory of Orbital Disease and Ocular Oncology, Shanghai, China

## Abstract

**Purpose:**

To evaluate the ocular pharmacokinetic properties of subretinal conbercept injection in vitrectomized rabbit eyes and to compare them with those by intravitreal injection.

**Methods:**

The following groups of New Zealand white rabbits received conbercept injections (0.5 mg/0.05 ml): a subretinal group (subretinal injection in vitrectomized eyes), an intravitreal group (intravitreal injection in vitrectomized eyes), and a control group (intravitreal injection in nonvitrectomized eyes). Drug concentrations in the aqueous humor (AH), the vitreous humor (VH), and the retina were measured by the enzyme-linked immunosorbent assay (ELISA), and pharmacokinetic parameters were calculated. Ophthalmic B-ultrasonography, electroretinogram (ERG), and hematoxylin and eosin (H&E) staining were performed to evaluate the safety of subretinal injection.

**Results:**

On the 28^th^ day after injection, the drug level in the subretinal group was significantly higher than that in the intravitreal group in the AH (0.90 ± 0.25 *μ*g/ml and 0.11 ± 0.07 *μ*g/ml and 0.11 ± 0.07 *P* < 0.001, respectively) and the VH (5.00 ± 3.86 *μ*g/ml and 0.11 ± 0.07 *μ*g/ml and 0.11 ± 0.07 *P* < 0.001, respectively) and the VH (5.00 ± 3.86 *P* < 0.001, respectively) and the VH (5.00 ± 3.86 *P* < 0.001, respectively) and the VH (5.00 ± 3.86

**Conclusions:**

Our study indicates that applying conbercept by subretinal injection can reduce the drug clearance rate and sustain a long maintenance period in ocular tissue, which suggests that subretinal conbercept injection may be a potentially valuable treatment option.

## 1. Introduction

Submacular hemorrhage (SMH) is a vision-threatening complication of neovascular age-related macular degeneration (AMD) and usually requires surgical management. After removing the blood clot, the postoperative visual outcome mainly depends on the extent of the underlying choroidal neovascularization (CNV) [1]. Vascular endothelial growth factor (VEGF) is crucial in angiogenesis because it is highly selective for endothelial cells and reaches its target by diffusion [2, 3], and the application of anti-VEGF agents during surgery could contribute to the prevention of CNV progression or recurrence [4, 5]. Although the current practice of intravitreal injection of anti-VEGF agents has produced an effective therapeutic response, research has shown that subretinal application can ensure delivery of the drug directly to the site of neovascularization and, thus may enhance the drug efficacy [6].

Intraoperative subretinal anti-VEGF injections have been used clinically in some cases, but the pharmacokinetic characteristics have not yet been determined. In addition to the progression of CNV, the damage of sensory retinal tissue by SMH has been attributed to the release of toxic substances, such as fibrin, iron, and hemosiderin [6]. To reduce the damage to the retina cells caused by bleeding, vitrectomy with subretinal anti-VEGF injection is usually required. Therefore, to better simulate the clinical treatment situation, we characterized the pharmacokinetics of conbercept as a model drug by subretinal application in a vitrectomized rabbit eye model and determined whether it could better reduce the clearance rate of conbercept compared with intravitreal injection.

Regarding the safety of subretinal application, a clinical study reported that no patients presented geographic atrophy or signs of retinal degeneration after subretinal anti-VEGF injection and the final visual improvement in most patients proves a tolerance of highly concentrated anti-VEGF agents in the subretinal space [6]. Additionally, the mechanical stress to the retinal tissue by the volume of the drug solution must be taken into consideration. Herein, we assessed the physiological and anatomical functions of the rabbit retina after subretinal injection of conbercept, providing essential evidence for safety evaluation.

## 2. Methods

### 2.1. Subjects

In this animal experiment, 18 healthy male New Zealand white rabbits (age 2 to 4 months; weight 2.5-3.0 kg) were maintained by the Zhejiang Institute of Medical Device Testing, Hangzhou, China. The study was approved by the Ethics Committee of the Zhejiang Institute of Medical Device Testing (permit number 2017002). The rabbits were divided into three groups (6 rabbits per group): a subretinal group (subretinal conbercept injection in vitrectomized eyes), an intravitreal group (intravitreal conbercept injection in vitrectomized eyes), and a control group (intravitreal conbercept injection in nonvitrectomized eyes).

### 2.2. Surgery and Intraocular Injections

The animals were anesthetized with an intravenous injection of 3% pentobarbital sodium (30 mg/kg; Sino Pharm Chemical Reagent Co., Ltd., China). Povidone-iodine was used for disinfection prior to surgery. The rabbit eyes underwent transconjunctival sutureless vitrectomy by using a 25-gauge trocar and cannula system (Constellation Vision System, Alcon Laboratories, Inc.) under general anesthesia. Vitrectomy was performed with one port used as the infusion cannula, one port for the surgical instrument, and a final port for illumination, and the vitreous was completely removed. The rabbits in the subretinal group received subretinal application of 0.5 mg/0.05 ml conbercept (Lumitin, Chengdu Kanghong Biotech Co., Ltd, P. R. China) at the inferior temporal side of the optic disc followed by fluid-air exchange, and the eyes were filled with balanced salt solution (BSS) tamponade. For the rabbits in the intravitreal group, fluid-air exchange and BSS tamponade were conducted, and then, the rabbits received intravitreal injection of conbercept (0.5 mg/0.05 ml). Transient hypotony was induced at the end of surgery to identify any potential bleeding sites. All operations were performed by the same vitreoretinal surgeon (Zhaoyang Wang). In the control group, the nonvitrectomized eyes were intravitreally injected with the same dose of conbercept (0.5 mg/0.05 ml).

### 2.3. Sample Collection

Aqueous humor (AH) and vitreous humor (VH) of the eyes were collected (0.05 ml) into sterile tubes 1 day, 3 days, 1 week, 2 weeks, and 4 weeks after injection. The retinal tissue of rabbits was obtained by a surgical separation 4 weeks after pars plana vitrectomy (PPV) and placed sterile icy phosphate buffer saline (PBS) containing cocktails before being fully ground. All samples were transported with liquid nitrogen and stored at −80°C until analysis.

### 2.4. Enzyme-Linked Immunosorbent Assay (ELISA)

Conbercept concentrations in the AH, the VH, and the retina were tested by ELISA, and the protocol was validated by the National Chengdu Center for Safety Evaluation of Drugs. The capture antibody was goat anti-human IgG-Fc (2.5 mg/ml; Bethyl, Montgomery, TX), and the detection antibody was goat anti-human VEGF receptor 2 conjugated with biotin (100 ng/ml; R&D, Minneapolis, MN). After incubation with streptavidin-conjugated horseradish peroxidase (HRP, R&D, Minneapolis, MN), tetramethylbenzidine (TMB) substrate was added. The reaction was stopped with dilute sulfuric acid. The optical density of each well was determined within 5 minutes by using a microplate reader set to 450 nm.

### 2.5. Electroretinogram (ERG)

For the functional evaluation, ERGs were measured in both eyes 1 week and 2 weeks after the subretinal conbercept injection. The animals were dark-adapted for at least 8 hours and anesthetized with 3% pentobarbital sodium. The pupils were fully dilated, and the eyes were topically anesthetized. The active electrode was placed on the cornea, the reference and ground electrodes were positioned subcutaneously on the forehead and on the ear of the rabbit, and the signals were recorded. Amplitudes of a-wave and b-wave (from the trough of the a-wave to the peak of the b-wave) were recorded in response to 10 cd *∗* s/m^2^ stimulation (dark-adapted and light-adapted).

### 2.6. H&E Staining

For anatomical evaluation, the rabbits that received subretinal injection were euthanized 1 week and 2 weeks after the subretinal conbercept injection. The globes were fixed for 48 hours with 4% paraformaldehyde, repeatedly washed with double distilled water, and then dissected vertically between the ora serrata regions, including the optic nerve head. The eyes were transferred to ethanol, embedded in paraffin, and then sectioned. The specimens were sectioned at 5 *μ*m, and every 10^th^ slide was stained with hematoxylin and eosin.

### 2.7. RPE Cell Viability

For safety assessment of the high concentration of conbercept, human retinal pigment epithelial cells (ARPE-19 cell line) were treated with 1×, 5×, and 10× concentrations of conbercept, according to the method described in the previous study [7]. The clinical dose of 0.5 mg conbercept was assumed to distribute equally throughout the 4 mL human vitreous and was defined as “1×.” A total of 5.0 × 10^3^ cells in 100 *μ*l of culture media (Dulbecco's modified Eagle's medium, Gibco, USA) per well were cultured in 96-well plates, and cell proliferation was tested with a Cell Counting Kit (CCK8, Dojindo, Japan).

### 2.8. Statistical Analysis

Statistical analysis was performed using SPSS software version 25.0 (IBM Corp., NY, US) and GraphPad Prism 7.0 (GraphPad Software, La Jolla, CA), and the results were presented as the mean ± standard deviation (SD). The *t*-test and the Mann–Whitney (*U* test) were used for inferential statistics. Quantitative data were checked for a normal distribution using the Shapiro–Wilk test. The level of significance was set to *P* < 0.05. In this study, conbercept was injected into the subretinal space and the drug penetrated from the retina to the VH and the AH, which suggested that the drug dynamics may not fit a typical compartment model and a noncompartmental model may be more suitable. The drug concentration-time data were analyzed by DAS 3.1.6 (Drug and Statistics, China), and the noncompartment model was adopted.

## 3. Results

### 3.1. Drug Concentration in the Samples

**Figure 1:**
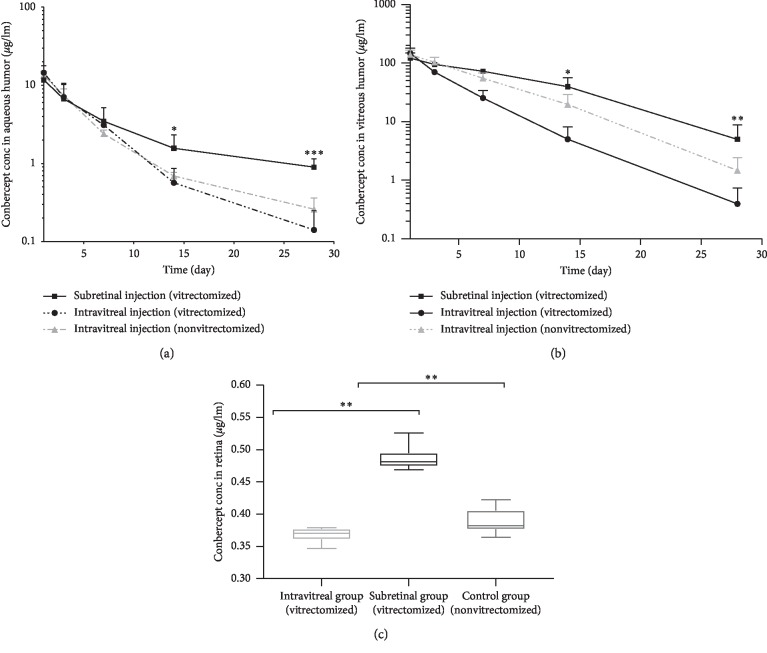
Concentrations of conbercept in the AH (a) and the VH (b) following administration of a single 0.5 mg dose to the left eyes in the subretinal group, the intravitreal group, and the intravitreal nonvitrectomized group. Conbercept concentration-time profiles for the AH and the VH are shown. The results are presented as the mean and upper error bar. A *t*-test was used for inferential statistics. (c) The concentration of conbercept in the retina was examined 4 weeks after injection. The results are presented as the mean and upper error bar. The *U* test was used for inferential statistics. ^*∗*^*P* < 0.05, ^*∗∗*^*P* < 0.01, and ^*∗∗∗*^*P* < 0.001.

According to subretinal or intravitreal application of conbercept, the rabbits were randomly assigned to 3 groups: a subretinal group (vitrectomized, *n* = 6), an intravitreal group (vitrectomized, *n* = 6), and a control group (intravitreal injection without PPV, *n* = 6). The concentrations of conbercept in the AH and the VH following administration of a single 0.5 mg dose to the left eyes in the groups were tested. The drug levels in the AH and the VH were highest at the 1^st^ time point (1 day) in the three groups, and then, the drug level in the subretinal group manifested a slower decline than that in the other two groups after the 3^rd^ (7 days) time point (Figures 1(a) and 1(b)). To compare the drug concentrations in the AH of the two vitrectomized groups, the conbercept levels in the subretinal group were 2.8 and 7.9 times higher than those in the intravitreal group on the 14^th^ day (1.57 ± 0.75 *μ*g/ml versus 0.57 ± 0.30 *μ*g/ml, *P*=0.013, respectively) and the 28^th^ day (0.90 ± 0.25 *μ*g/ml versus 0.11 ± 0.07 *μ*g/ml, *P* < 0.001, respectively) after injection, and the drug levels in the VH of the subretinal group were also significantly higher than those in the intravitreal group on the 14^th^ day (39.52 ± 16.50 *μ*g/ml versus 5.05 ± 3.10 *μ*g/ml, *P*=0.001, respectively) and the 28^th^ day (5.00 ± 3.86 *μ*g/ml versus 0.40 ± 0.34 *μ*g/ml, *P*=0.016, respectively). For intravitreal injection, the drug level in the AH of the nonvitrectomized group was also significantly higher than that in the vitrectomized group (0.26 ± 0.10 *μ*g/ml versus 0.11 ± 0.07 *μ*g/ml, *P*=0.016, respectively; Figure 1(a)) 4 weeks after the injection, and the drug level was also significantly different in the VH (1.48 ± 0.97 *μ*g/ml versus 0.40 ± 0.34 *μ*g/ml, *P*=0.028, respectively; Figure 1(b)).

The drug levels in the retina were 0.49 ± 0.02 *μ*g/ml, 0.37 ± 0.01 *μ*g/ml, and 0.39 ± 0.02 *μ*g/ml in the subretinal group, the intravitreal group, and the control group, respectively (Figure 1(c)). The remaining drug concentration in the retina was significantly higher after subretinal application than in the intravitreal group (*P*=0.002) and the control group (*P*=0.002; Figure 1(c)).

### 3.2. Ocular Pharmacokinetics of Conbercept

The elimination pharmacokinetic parameters of conbercept are summarized in Table 1. The drug levels in the AH and the VH had a mean terminal half-life of 9.88 days and 6.14 days by subretinal application, which was significantly longer than the mean half-life by intravitreal application in the AH (3.34 days, *P*=0.004; Table 1) and the VH (3.12 days, *P*=0.002; Table 2), respectively. However, the half-lives in the intravitreal and control groups were not statistically significant in the AH (*P*=0.304; Table 1). The last area under the first moment curve (AUMC_0-t_) of the AH in the subretinal group was substantially larger than that in the intravitreal group (1.99 times larger; if the predicted AUMC infinity was used for the comparison, the total drug exposure was 3.48 times larger; *P*=0.004 and *P*=0.009, respectively). The area under the curve (AUC_0-t_) and the AUMC_0-t_ of the VH in the subretinal group were also significantly larger than those in the intravitreal group (both *P* < 0.001). The last mean residence time (MRT_0-t_) was significantly longer by subretinal injection than by intravitreal injection in the AH (*P* < 0.001) and the VH (*P* < 0.001). The intergroup difference in MRT_0-t_ between the intravitreal and control groups was not significantly different.

### 3.3. Safety Assessment (Ophthalmic B-Ultrasonography, ERG, H&E Staining, and Cell Viability)

**Figure 2:**
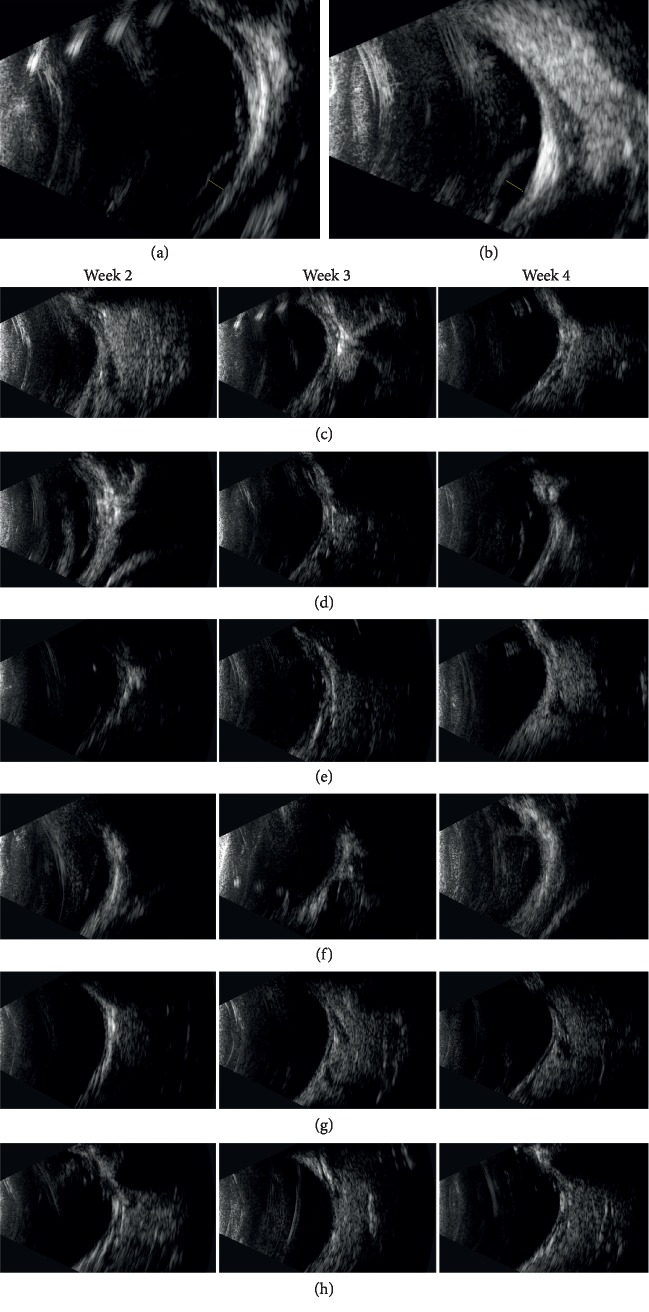
(a, b) A slight uplift of the retina caused by the volume of the drug solution was observed in two eyes (two rabbits) of the subretinal group 24 hours after injection. The height of the uplift was measured (the yellow straight line). (c–h) B-ultrasonography of the six eyes (six rabbits) in the subretinal group 2 to 4 weeks after conbercept injection. The slight uplift of the retina reverted to a flat state in (e) and (f). No retinal detachment or vitreous hemorrhage was observed.

**Figure 3:**
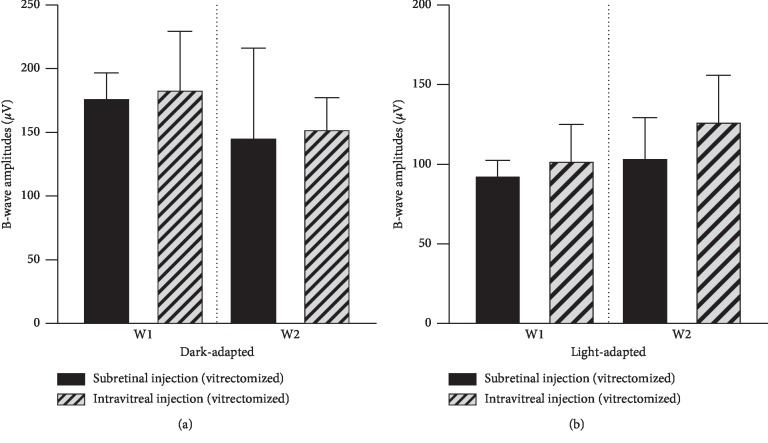
Dark-adapted (a) and light-adapted (b) ERGs. The b-wave amplitudes in response to 10 cd *∗* s/m^2^ stimulation of the dark-adapted and light-adapted eyes 1 week (W1) and 2 weeks (W2) after injection into vitrectomized eyes.

**Figure 4:**
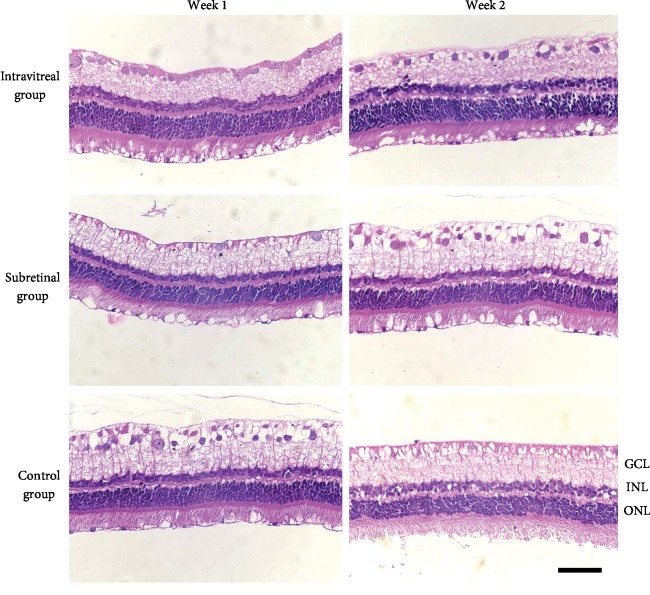
Sections of the rabbit retina labeled with hematoxylin and eosin: eyes from the subretinal group (subretinal injection into vitrectomized eyes), the intravitreal group (intravitreal injection into vitrectomized eyes), and the control group (intravitreal injection into nonvitrectomized eyes) with conbercept injection 1 week and 2 weeks postoperatively. The histological examinations show the complete structure of all retinal layers in the eyes of the 3 groups. No signs of atrophy, disorganization, cell loss, or hypocellularity were observed. The scale bar is 50 *μ*m.

**Figure 5:**
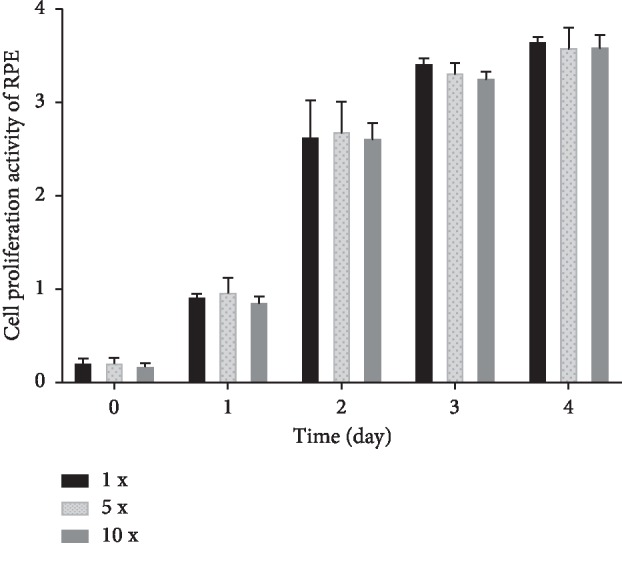
RPE were treated with 1×, 5×, and 10× concentrations of conbercept, and cell proliferation was examined.

The ophthalmic B-ultrasonography of the vitrectomized rabbit eyes in the subretinal group is shown in Figures 2(a)–2(h) for mechanical safety assessment. A slight uplifting of the retina caused by the volume of the drug solution was observed in two eyes (two rabbits) 24 hours after subretinal application. The eyes were uplifted by 1.25 mm (Figure 2(a)) and 1.75 mm (Figure 2(b)) and reverted to a flat state (Figures 2(e) and 2(d), respectively) during the next four weeks after injection. No retinal detachment or vitreous hemorrhage was observed (Figures 2(c)–2(h)). The b-wave amplitudes in response to 10 cd *∗* s/m^2^ stimulation of dark-adapted and light-adapted eyes at 1 week and 2 weeks after the injection were not significantly different between the subretinal and intravitreal groups (Figures 3(a) and 3(b); Table 3), and no evidence of retinal functional damage was observed after subretinal application of conbercept. The histological examination by H&E staining revealed complete preservation of all retinal layers in both the subretinal injection eyes and the intravitreal injection eyes (Figures 4(a)–4(f)). No signs of atrophy, disorganization, cell loss, or hypocellularity were observed in either the inner or outer retina of the rabbit eyes. Additionally, the proliferation of ARPE-19 cells treated with 1×, 5×, and 10× concentrations of conbercept showed no intergroup differences (Figure 5).

## 4. Discussion

Conbercept is a recombinant fusion protein with a high binding affinity for all isoforms of VEGF and the ability to strongly inhibit choroidal neovascularization. Conbercept is composed of human VEGF receptor 1 (domain 2), VEGF receptor 2 (domains 3 and 4), and the Fc fragment of human IgG1 [8]. This large fusion protein is unlikely to cause systemic complications because its permeability through blood-ocular barriers is hindered by its 143 kDa molecular size [8, 9]. Therefore, subretinal injection of this large protein is relatively safe. Subretinal injection was first adopted using a transretinal 41-gauge subretinal flexible cannula (DORC, Zuidland, the Netherlands) for more effective delivery of rtPA into the subretinal space, allowing it to directly target subretinal hemorrhage for maximal clot lysis [10].

As anti-VEGF agents have been used to manage massive SMH for the past few years, coapplication of anti-VEGF agents with vitrectomy has been advocated for visual improvement. Compared with interventions for removing submacular blood, including pneumatic displacement of the clot, recombinant tissue plasminogen activator (rtPA) injection for clot dissolution, and transplantation of RPE or a choroidal patch, anti-VEGF administration [11] is the only method for angiogenesis prevention. Polypoidal choroidal vasculopathy (PCV) was first regarded as a subtype of neovascular AMD, and its pathology is characterized by type 1 neovascularization with aneurysmal dilations known as polyps; however, controversy remains because some hallmarks of AMD are absent in PCV [12]. Both AMD and PCV can lead to severe submacular hemorrhage, and anti-VEGF treatment is effective against these two vascular diseases. According to previous studies, anti-VEGF is considered the first-line therapy for neovascular AMD [7], and significant improvement of the mean best-corrected visual acuity (BCVA) has been observed in PCV after anti-VEGF injection [13]. Another study reported a loss of acuity over time after PPV with subretinal rtPA injection and gas tamponade displacement of SMH, and anti-VEGF therapy may benefit the final visual function [14]. Therefore, the persistent anti-VEGF effect is essential for preventing hemorrhage recurrence in the submacular space or vitreous cavity. For patients with AMD, any delay in injection administration during the maintenance phase might not result in a favorable visual outcome and visual function may worsen if treatment is administered discontinuously [3, 15, 16].

The effects of intravitreal conbercept injection are known to be relatively transient, with a reported AH half-life of 2.40 days after a 0.5 mg dose of intravitreal injection in nonvitrectomized rabbits [7]. Some published clinical trials reported subretinal injection of anti-VEGF drugs during vitrectomy surgery for the treatment of AMD and SMH [1, 2]. Therefore, to better simulate the clinical treatment situation, we performed a pharmacokinetic study using a vitrectomy rabbit eye model. The rabbit has been reported to be a useful animal model for studying intravitreal pharmacokinetics and exhibits good correlation and comparability to humans [17]. A previous animal study demonstrated that the overall intraocular pharmacokinetic parameters of intravitreal ranibizumab injection in vitrectomized eyes were similar to the properties in nonvitrectomized rabbit eyes [18], in accordance with the results in our study (Tables 1 and 2). As the suggested therapy for conbercept was three sequential monthly injections in nonvitrectomized eyes, patients who undergo vitrectomy and receive one intravitreal conbercept injection will probably require another injection one month after the last injection [19, 20]. Therefore, according to the results of our study that subretinal injection provides a long drug maintenance period, the frequency of anti-VEGF treatment might be decreased based on the prolonged half-life and MRT_0-t_ of the AH and the VH (Tables 1 and 2).

Since the drug can pass through the retina into the vitreous cavity immediately after subretinal application, the drug concentration under the retina cannot be accurately measured or estimated. A previous in vitro study compared the safety profiles of anti-VEGF agents, including ranibizumab, bevacizumab, aflibercept, and ziv-aflibercept, on human ARPE-19 cells in culture [21]. The ARPE-19 cells were exposed to the four types of anti-VEGF drugs at 1/2×, 1×, 2×, and 10× clinical concentrations for 24 hours, and cell viability was evaluated. To assess the safety of the high drug concentration in the retina in the current study, the method mentioned above was adopted and the results showed that cell viability was not significantly affected. Regarding the mechanical stress of the retina, two of the rabbits (33.3%) were observed to have a slight uplift due to the volume of the drug solution. However, the results of the in vivo experiments (including B-ultrasonography, ERG, and H&E staining) in the present study showed no influence on the physiological and anatomical functions of the retina after subretinal conbercept injection. Therefore, subretinal conbercept injection may be safe, and the drug may directly act on CNV to prevent postoperative rebleeding [14, 22].

In conclusion, we performed a pharmacokinetic analysis of conbercept (0.5 mg/0.05 ml) by subretinal application. Subretinal conbercept injection decreased the clearance rate of conbercept and was sustained for a long maintenance period. The prolonged anti-VEGF effect by intraoperative subretinal conbercept injection may be beneficial for preventing angiogenesis and postoperative hemorrhage.

## Figures and Tables

**Table 1:** Pharmacokinetic parameters of the conbercept model in aqueous humor.

Parameters	Intravitreal injection (vitrectomized; *n* = 6)	Subretinal injection (vitrectomized; *n* = 6)	Intravitreal injection (nonvitrectomized; *n* = 6)	*P* value
*C*_max_ (*μ*g/ml)^†^	14.49 ± 3.40	11.75 ± 3.38	13.43 ± 1.59	*P*=0.192
*T*_max_ (days)^††^	1.00 ± 0.00	1.00 ± 0.00	1.00 ± 0.00	*P*=1.000
Half-life (days)^†^	3.34 ± 0.58	9.88 ± 6.48	4.30 ± 1.78	*P* = 0.004^*∗∗*^
AUC_0-t_ (*μ*g day/ml)^†^	66.81 ± 18.42	79.56 ± 30.58	64.76 ± 6.85	*P*=0.402
AUC_0-∞_ (*μ*g day/ml)^†^	67.02 ± 18.36	89.38 ± 25.60	65.49 ± 7.07	*P*=0.110
AUMC_0-t_ (*μ*g day^2^/ml)^††^	310.76 ± 96.32	618.20 ± 239.12	332.31 ± 32.21	*P*=0.004^*∗∗*^
AUMC_0-∞_ (*μ*g day^2^/ml)^†^	317.83 ± 95.23	1105.85 ± 474.77	359.66 ± 59.79	*P*=0.009^*∗∗*^
MRT_0-t_ (days)^†^	4.62 ± 0.49	7.85 ± 0.89	5.15 ± 0.41	*P* < 0.001^*∗∗∗*^
MRT_0-∞_ (days)^†^	4.72 ± 0.50	13.10 ± 6.94	5.49 ± 0.68	*P*=0.032^*∗*^

*C*_max_, maximum concentration; *T*_max_, the time at which the concentration was maximum; AUC, area under the curve; AUMC, area under the first moment curve; MRT, mean residence time.^†^*t*-test: comparison of the parameters in the intravitreal group (vitrectomized) and the subretinal group (vitrectomized).^††^*U* test: comparison of the parameters in the intravitreal group (vitrectomized) and the subretinal group (vitrectomized). The parameters were calculated by a noncompartmental model, and ^*∗*^*P* < 0.05, ^*∗∗*^*P* < 0.01, and ^*∗∗∗*^*P* < 0.001 were considered to indicate significance.

**Table 2:** Pharmacokinetic parameters of the conbercept model in vitreous humor.

Parameters	Intravitreal injection (vitrectomized; *n* = 6)	Subretinal injection (vitrectomized; *n* = 6)	Intravitreal injection (nonvitrectomized; *n* = 6)	*P* value
*C*_max_ (*μ*g/ml)^††^	147.16 ± 34.44	121.63 ± 27.59	140.45 ± 25.81	*P*=0.394
*T*_max_ (days)^††^	1.00 ± 0.00	1.33 ± 0.82	1.33 ± 0.82	*P*=0.699
Half-life (days)^†^	3.12 ± 0.45	6.14 ± 1.69	4.12 ± 0.98	*P*=0.002^*∗∗*^
AUC_0-t_ (*μ*g day/ml)^†^	628.08 ± 145.71	1318.15 ± 243.56	1039.69 ± 243.99	*P* < 0.001^*∗∗∗*^
AUC_0-∞_ (*μ*g day/ml)^†^	629.48 ± 145.76	1384.53 ± 272.42	1053.33 ± 254.53	*P* < 0.001^*∗∗∗*^
AUMC_0-t_ (*μ*g day^2^/ml)^†^	2653.01 ± 891.88	10633.25 ± 3431.12	6447.03 ± 2108.65	*P* < 0.001^*∗∗∗*^
AUMC_0-∞_ (*μ*g day^2^/ml)^†^	2699.37 ± 901.33	13171.78 ± 4838.04	6927.17 ± 2518.34	*P* < 0.001^*∗∗∗*^
MRT_0-t_ (days)^†^	4.15 ± 0.55	7.90 ± 1.25	6.09 ± 0.60	*P* < 0.001^*∗∗∗*^
MRT_0-∞_ (days)^†^	4.22 ± 0.58	9.25 ± 1.89	6.42 ± 0.86	*P* < 0.001^*∗∗∗*^

*C*_max_, maximum concentration; *T*_max_, the time at which the concentration was maximum; AUC, area under the curve; AUMC, area under the first moment curve; MRT, mean residence time.^†^*t*-test: comparison of the parameters in the intravitreal group (vitrectomized) and the subretinal group (vitrectomized).^††^*U* test: comparison of the parameters in the intravitreal group (vitrectomized) and the subretinal group (vitrectomized). The parameters were calculated by the noncompartmental model, and ^*∗*^*P* < 0.05, ^*∗∗*^*P* < 0.01, and ^*∗∗∗*^*P* < 0.001 were considered to indicate significance.

**Table 3:** Electroretinography of vitrectomized eyes.

Parameters	Dark-adapted		*P* value	Light-adapted		*P* value
	Intravitreal injection	Subretinal injection		Intravitreal injection	Subretinal injection	
Week 1	154.96 ± 23.82	146.57 ± 69.90	*P*=0.786	104.74 ± 24.59	127.70 ± 28.07	*P*=0.163
Week 2	184.23 ± 45.01	177.66 ± 19.18	*P*=0.749	93.76 ± 8.63	101.88 ± 25.71	*P*=0.480

Averaged values ± standard deviations of the b-wave amplitudes (*μ*V). The *t*-test was used to compare b-wave amplitudes in the intravitreal group (vitrectomized) and the subretinal group (vitrectomized), and ^*∗*^*P* < 0.05 was considered to indicate significance.

## Data Availability

The data used to support the findings of this study are included within the article.
